# CO_2_ exposure as translational cross-species experimental model for panic

**DOI:** 10.1038/tp.2016.162

**Published:** 2016-09-06

**Authors:** N K Leibold, D L A van den Hove, W Viechtbauer, G F Buchanan, L Goossens, I Lange, I Knuts, K P Lesch, H W M Steinbusch, K R J Schruers

**Affiliations:** 1 Department of Psychiatry and Neuropsychology, Maastricht University, Maastricht, The Netherlands; 2 School for Mental Health and Neuroscience, Maastricht University, European Graduate School of Neuroscience, Maastricht, The Netherlands; 3 Department of Neurology, Yale University, New Haven, CT, USA; 4 Division of Molecular Psychiatry, Laboratory of Translational Neuroscience, Center of Mental Health, University of Wuerzburg, Wuerzburg, Germany; 5 Department of Neurology, University of Iowa, Iowa City, IA, USA; 6 Center for the Psychology of Learning and Experimental Psychopathology, Department of Psychology, University of Leuven, Tiensestraat, Leuven, Belgium

## Abstract

The current diagnostic criteria of the Diagnostic and Statistical Manual of Mental Disorders are being challenged by the heterogeneity and the symptom overlap of psychiatric disorders. Therefore, a framework toward a more etiology-based classification has been initiated by the US National Institute of Mental Health, the research domain criteria project. The basic neurobiology of human psychiatric disorders is often studied in rodent models. However, the differences in outcome measurements hamper the translation of knowledge. Here, we aimed to present a translational panic model by using the same stimulus and by quantitatively comparing the same outcome measurements in rodents, healthy human subjects and panic disorder patients within one large project. We measured the behavioral–emotional and bodily response to CO_2_ exposure in all three samples, allowing for a reliable cross-species comparison. We show that CO_2_ exposure causes a robust fear response in terms of behavior in mice and panic symptom ratings in healthy volunteers and panic disorder patients. To improve comparability, we next assessed the respiratory and cardiovascular response to CO_2_, demonstrating corresponding respiratory and cardiovascular effects across both species. This project bridges the gap between basic and human research to improve the translation of knowledge between these disciplines. This will allow significant progress in unraveling the etiological basis of panic disorder and will be highly beneficial for refining the diagnostic categories as well as treatment strategies.

## Introduction

Currently, the diagnoses of psychiatric disorders are based on the Diagnostic and Statistical Manual of Mental Disorders (DSM),^1^ which specifies the minimum number and duration of symptoms that must be present for a particular diagnosis. The symptom-based categories of DSM have been the gold standard for many years; however, the heterogeneity and symptom overlap of many mental disorders challenge this categorical approach. Therefore, a new framework toward a novel dimensional classification based on integrating behavior and neurobiological measures has been initiated by the US National Institute of Mental Health, the research domain criteria.^2^ It is expected that combining self-reports with assessment of behavior, genetics and neural circuitry will lead to a more etiology-based diagnosis rather than a mainly symptom-driven one, ultimately leading to improved treatments in the long term.

Although behavioral and genetic assessments are technically feasible in humans, the potential to study neural circuitries is limited. Therefore, a vast proportion of research into the basic neurobiology of human psychiatric conditions is carried out in other species, making use of animal (mainly rodent) models of psychopathology. A major challenge in this respect is the translation of data from research in animals to humans and, ultimately, patients with a psychiatric disorder. To maximize the efficacy of translation, the rodent model should resemble the aspects of the disorder in humans as much as possible.

To judge the degree of an animal model reflecting the disorder of interest, the following criteria are commonly used:^3^ (1) face validity, indicating the resemblance between the behavior in the model and the symptoms of the disorder, (2) predictive validity, referring to the degree the impact on the model predicts the outcome in the disorder and (3) construct validity, referring to the degree of similarity between the etiological mechanisms underlying behavior in the rodent model and in the disorder. In the past few years, important progress has been made in psychiatry research using well-validated animal models. For instance, the assessment of prepulse inhibition, a measure of sensorimotor gating, in rodents, has led to insight in sensory processing in humans and has contributed to the knowledge of the neuronal dysfunctions in psychiatric disorders such as schizophrenia.^4^ Such an example is rare in psychiatry, however.

Panic attacks (PAs) are a common psychopathological phenomenon throughout many psychiatric disorders, but most notably they are the core feature of panic disorder (PD).^1^ It has been well established that PAs can be reliably provoked in the laboratory using a brief inhalation of 35% CO_2_.^5, 6, 7, 8, 9, 10, 11^ Originally, it was believed that the hyper-reactivity to such a CO_2_ inhalation was specific to PD.^12, 13, 14, 15^ However, a recent series of studies showed that PAs can also be reliably induced in healthy subjects, depending on the concentration of CO_2_ and personal sensitivity.^6, 16^ The finding that panic can thus be studied in healthy volunteers is important as major confounders that are present in patient populations, such as comorbidity or the use of medication, can be avoided. In addition, the observation that patients are more sensitive to CO_2_ than healthy individuals demonstrates the existence of a continuous CO_2_ sensitivity spectrum based on common fundamental mechanisms that are present in every individual. Human studies showed that PD has a heritability of ~40%^17^ and that the individual differences in the sensitivity to CO_2_ can be, in part, attributed to genetic factors.^18, 19, 20^ A higher concordance was found for monozygotic twins compared with dizygotic ones,^21^ and healthy first-degree relatives of PD patients have an elevated sensitivity to CO_2_, which is intermediate between healthy individuals without a familial history of PD and PD patients.^22, 23, 24^ In recent rodent studies, inspired by the use of CO_2_ as human experimental model for panic, animals were exposed to concentrations of up to 20% CO_2_ in order to investigate the basic mechanisms of fear.^25, 26, 27, 28, 29^ Quantitative genetic research in rodent unrelated individuals, sibs and half-sibs support a genetic contribution in the sensitivity to CO_2_.^29^ Other seminal discoveries were made such as the essential role of pH-sensitive ion channels^27^ and that of orexin^26^ in CO_2_-induced rodent fear behavior. The use of experiments that represent the human models as closely as possibly^27, 29, 30^ is an important element in the application of these basic findings to the study of fear in humans. However, translation is commonly hampered by the fact that rodent and human studies use very different outcome measures. Most human panic studies rely on self-reports of fear and discomfort levels, whereas animal studies primarily use behavioral assays to assess the fear and distress evoked by CO_2_ in relationship to neurobiological alterations. Additional outcome measures in both species might allow a better comparison of the data.

In the present paper, we present data from three translational studies applying CO_2_-induced fear in rodents, healthy human individuals and PD patients. Apart from the traditional outcome measures (behavioral tests in mice and symptom reports in humans), we present data from comparable physiological measurements in both species. We thus aim to present a translational experimental panic model combining three samples within one project.

## Materials and methods

### Study 1: mice

#### Animals

In total, 20 male wild-type C57BL/6 mice were housed within a temperature-controlled environment and under a reversed day/night cycle (12 h dark/12 h light cycle). Animals had access to standard rodent chow and water *ad libitum*. For behavioral testing, mice were assigned to either CO_2_ exposure (*n=*10) or air exposure (*n=*10). The physiological response to both room air and CO_2_ exposure was tested in the same animals (*n=*20). Sample sizes were calculated using G*Power 3.1.9.2 (ref. 31) and the parameters alpha=0.05 and power=0.8. For the between-subject comparison, the calculation was based on independent *t*-test (two-tailed, *d*=1.4 based on previous behavioral experiments) and the within-subject comparison on two dependent means (two-tailed, *d*=0.7 based on previous experiments). Animals were assigned to groups in a semi-randomized manner by an investigator not directly involved in the testing and ensuring a balanced age distribution among groups. Testing was performed from a mean age of 58 days onward under low-light conditions by an investigator aware of experimental condition to ensure exposure to the right CO_2_ concentration. Using a computer program the order of testing animals was randomized and kept for all experiments. Behavioral analysis of video files was performed by an investigator blinded to the experimental conditions. All procedures and experiments were approved by the Institutional Animal Care and Use Committee at Yale University, New Haven, CT, USA. Animals were handled in accordance with the institutional guidelines.

#### CO_2_ exposure and behavioral testing

**Open field test:** For the first behavioral test, the open field test (OFT), a transparent Plexiglas square chamber (60 × 60 × 40 cm), covered with a clear lid, was used. The floor was subdivided into a 30 × 30 cm central zone, 15 × 15 cm corners and 30 × 15 cm walls. Depending on the experimental group, the chamber was pre-filled with 9% CO_2_ (normoxic mixture; compressed air tanks, Airgas East, Cheshire, CT, USA) or air. Gas infusion ports and two CPU fans (21 dB, ebm-papst, St Georgen, Hungary) to ensure a homogenous concentration of CO_2_ throughout the chamber were located on the upper part of the box to avoid blowing, which may be aversive to mice. The concentration of CO_2_ was constantly controlled using a digital CO_2_ meter (30% CO_2_ Sampling Data Logger, CO_2_ meter, Ormond Beach, FL, USA). After pre-filling the chamber with gas, mice were placed in the center of the chamber and were allowed to explore the chamber for 20 min.^27^ Movements were recorded automatically with a computerized system (Ethovision Pro, Noldus, Wageningen, the Netherlands). The number of fecal pellets was scored at the end of each trial by the experimenter. The box was cleaned with 70% ethanol between trials to avoid olfactory cues.

**Two-chamber test:** Three days after the OFT, animals were tested in the two-chamber test (TCT). The testing apparatus consisted of two chambers (each 30 × 30 cm with 40 cm high walls), which were connected by an open door (3.5 × 3.5 cm) to allow free crossing. Each chamber had a gas infusion port and a CPU fan (21 dB, ebm-papst) to ensure a homogenous gas concentration throughout the entire part. For mice subjected to CO_2_ exposure, one chamber was pre-filled with CO_2_ and the other one with room air. Gas concentrations were controlled continuously using a digital CO_2_ meter (30% CO_2_ Sampling Data Logger, CO_2_ meter; reaching a steady state of 9% and 2% CO_2_, respectively). For mice assigned to air exposure, air was used in both chambers. Side of CO_2_ administration and side in which the mouse was placed were randomized using a computer program. Movements were recorded automatically with a computerized system (Ethovision Pro, Noldus) for a period of 10 min. The number of fecal pellets was scored at the end of each trial by the experimenter. Ethanol (70%) was used to clean the chambers between trials to avoid olfactory cues.

**CO_2_-evoked freezing:** Freezing was scored in both behavioral tests by a trained observer and was defined as absence of any movements apart from respiration.

#### Breathing and cardiovascular recordings

After completion of the behavioral tests, breathing and the cardiovascular response were assessed in all animals. Mice were placed into a custom-made whole-body recording chamber (Plexiglas, 350 cm^3^). Animals were habituated to the chamber for 30 min (with room air infusion). Then, the physiological response was measured in a fixed order: room air exposure for 20 min and subsequently 9% CO_2_ for 10 min. Flow rates (0.4 l min^−1^) were controlled with a digital flowmeter (WU-32446-33, Cole-Parmer, Hoffman Estates, IL, USA).

Breathing and cardiovascular recordings were obtained non-invasively and simultaneously. Breathing was assessed using a low-volume pressure transducer (DC002NDR5; Honeywell International, Minneapolis, MN, USA) that was fitted to the recording chamber. Breathing-induced pressure changes were calibrated with 150 pulses per min, each 300 μl. Relative humidity (HIH-4602-A sensor; Honeywell International, Minneapolis, MN, USA) and ambient temperature (BAT-12 microprobe, Physiotemp Instruments, Clifton, NJ, USA) were measured continuously within the recording chamber. Electrodes for electrocardiogram measurements were placed on both sides of the shaved thorax and connected to an amplifier (Model 440 Instrumentation Amplifier, Brownlee Precision, San Jose, CA, USA). Blood pressure was not assessed as common methods such as the use of a tail cuff do not allow monitoring in freely moving animals or require prior surgery as it is the case in telemetry. Animal temperature was measured rectally immediately after completion of the recording (BAT-12 microprobe, Physiotemp Instruments). All signals were digitized with an analog-to-digital converter (PCI-6221 or USB-6008 National Instruments, Austin, TX, USA) and were displayed in a custom-written acquisition program in Matlab (version R2011b, Mathworks, Natick, MA, USA).

### Study 2: healthy volunteers

#### Participants

In total, 136 adult healthy volunteers (mean age 22.81 years, s.d.=8.89, 44 males) were recruited via advertisements at Maastricht University, the Netherlands (part of a previous sample^32^ extended with new participants (*n=*79)). The subsamples did not differ significantly regarding demographics (age and sex), psychometric scales (fear and panic scores) or physiology (independent samples *t*-test). As the smallest effects were expected for 0% CO_2_ compared with 9% CO_2_ (see ‘CO_2_ inhalation and fear/panic scores' for details about the used concentrations), the sample size calculation was based on this comparison using a dependent means test in G*Power 3.1.9.2 (ref. 32) (two-tailed, alpha=0.05, power=0.8, *d*=0.25 based on a previous pilot study assessing the cardiovascular effects to CO_2_, leading to a total sample size of 128). Before participating, eligibility was confirmed using a medical examination and a semi-structured psychiatric interview (including the Mini International Neuropsychiatric interview^33^). Pre-defined exclusion criteria were current or past cardiovascular or pulmonary disease, hypertension (systolic/diastolic >170/100 mmHg), familial or personal history of cerebral aneurysm, excessive smoking (>15 cigarettes per day), pregnancy, epilepsy, use of psychotropic medication or adrenergic receptor blockers and a first-degree relative with PD. All participants gave written informed consent before the study, which was approved by the Medical Ethics Committee of Maastricht University and the Maastricht University Hospital.

#### CO_2_ inhalation and fear/panic symptom scores

After confirming eligibility, participants took a double vital capacity breath of four CO_2_ concentrations according to a double-blind, randomized, cross-over design: 0, 9, 17.5 and 35% CO_2_ (normoxic gas mixture; premixed gas tanks obtained from Nederlandse Technische Gasmaatschappij, Landgraaf, the Netherlands). A computer program determined a random order for each individual, which was verified by an independent investigator to ensure that each concentration was applied once. The double vital capacity breath of 35% CO_2_ induces a condition complying with the formal criteria of panic in the current psychiatric nosology.^16^ Each inhalation was performed according to a standardized protocol in our laboratory.^6, 16, 32^ More specifically, after being seated in an armchair, a nasal–oral mask was fixed to the participant's head. The vital capacity of a double breath was measured using a digital flowmeter. All participants were told that the subsequent CO_2_ inhalation might cause some effects, ranging from vague sensations up to fear. However, all effects would be short-lasting. Subsequently, participants took a double vital capacity breath of one CO_2_ mixture. Participants were motivated to inhale at least 80% of the vital capacity. Inhalations took place on four separate days, each day at the same time for each participant. Participants were instructed to refrain from caffeine-containing beverages on the inhalation days.

Presence and intensity of fear and panic symptoms were obtained twice: immediately before the inhalation, patients were asked to rate their sensations at that particular moment and after the inhalation to rate the sensations at the worst moment of the inhalation. Feelings of fear were evaluated using the Visual Analog Scale for fear (VAS-F), a horizontal line of 100 mm length that ranges from 0 (not at all) to 100 (the worst imaginable). Panic symptoms were assessed using the Panic Symptom List (PSL), consisting of the 13 DSM PA symptoms and ranging from 0 (absent) to 4 (very intense).

#### Breathing and cardiovascular recordings

Physiological parameters were measured throughout all four CO_2_ inhalation using a computerized system (Carbon Dioxide Tolerance Tester, Maastricht Instruments, Maastricht, the Netherlands) as previously described.^32^ A soft plastic nasal–oral mask was fixed to the participant's head and was connected to a capnograph device (Medair, Delsbo, Sweden) to measure the respiration rate. A finger cuff, connected to a cardiovascular monitor (Nexfin, Bmeye, Amsterdam, the Netherlands), was fixed to the middle finger of the non-dominant hand to assess heart rate and blood pressure (sampling rate 2 Hz). All measurements were acquired with a custom-made software (IDEEQ, Maastricht Instruments).

### Study 3: PD patients

#### Participants

Ninety-eight adult patients with PD (mean age 35.21 years, s.d.=11.65, 63 males) from the outpatient setting of the Academic Anxiety Centre, Maastricht, the Netherlands, voluntarily participated in this study. To compare the CO_2_-induced effects in PD patients with healthy individuals, a test for independent means was used in G*Power 3.1.9.2 (ref. 32) (two-tailed, alpha=0.05, power=0.8, *d*=0.4 based on a previous pilot study). PD (with or without agoraphobia) as the main diagnosis was established via a semistructured psychiatric interview (including the Mini International Neuropsychiatric interview^33^) by an experienced psychiatrist. In addition, a medical examination took place. Pre-defined exclusion criteria were current or past cardiovascular or pulmonary disease, hypertension (systolic/diastolic >170/100 mmHg), familial or personal history of cerebral aneurysm, pregnancy and epilepsy. All participants provided written informed consent. The study was approved by the Medical Ethics Committee of Maastricht University and the Maastricht University Hospital.

#### CO_2_ inhalation and fear/panic symptom scores

Patients took a single vital capacity breath of 35% CO_2_ (premixed gas tanks obtained from Nederlandse Technische Gasmaatschappij) according to a repeatedly used and standardized protocol in our laboratory.^11, 34^ We previously showed that this inhalation provokes the fear and panic symptoms in patients resembling a real-life PA,^11^ whereas in healthy individuals a double breath induces qualitatively comparable effects.^16^ Patients were seated in an armchair and instructed into the use of a self-administering nasal–oral mask. The vital capacity of a single breath was measured using a digital flowmeter. Then, the patients were informed that the subsequent CO_2_ inhalation might cause some effects, ranging from vague sensations up to fear. However, all effects would be short-lasting. Patients took a single vital capacity breath of 35% CO_2_ and were motivated to inhale at least 80% of the previously measured vital capacity. Patients were instructed to refrain from caffeine-containing beverages on the inhalation day.

Likewise to the assessment in healthy volunteers, fear and panic symptom scores were obtained immediately before and after the CO_2_ inhalation. The VAS-F was used to assess fear (ranging from 0, not at all, to 100, the worst imaginable) and the PSL for assessing the 13 symptoms of a PA (ranging from 0, absent, to 4, very intense).

#### Breathing and cardiovascular recordings

As in healthy individuals, physiological recordings were obtained using a computerized system (Carbon Dioxide Tolerance Tester, Maastricht Instruments) throughout the entire procedure. Measuring the respiration rate implies using a mask fixed to the head, which was not tolerated by the patients in a pilot study in our laboratory. Therefore, we only recorded heart rate and blood pressure, which we measured using a finger cuff that was fixed to the middle finger of the non-dominant hand (sampling rate 2 Hz). The finger cuff was connected to a cardiovascular monitor (Nexfin, Bmeye). All measurements were collected and displayed in the custom-made software (IDEEQ, Maastricht Instruments).

### Data and statistical analysis

For all analyses, statistical significance was set at *P<*0.05 (two-tailed). Assumptions for tests such as normal distribution, homoscedasticity and (in)dependence of observations were checked and met. Analyses were carried out using the software R (version 3.1.1, 2014, R Development Core Team, Vienna/Austria) or the Statistical Package for the Social Sciences (SPSS 20.0.0.1 for Mac; SPSS, Chicago, IL, USA). Values are presented as means+s.e.m.

#### Animal behavioral data

Rodent behavior was analyzed using univariate analysis of variance or independent *t*-tests for unequal variances, when appropriate. Regarding the TCT, within a group, repeated measures analysis was applied. To take freezing behavior into account, the ratio for freezing in a particular chamber divided by the time spent in that chamber was calculated.

#### Human symptom reports

In humans, self-reports were analyzed in terms of change in fear and panic symptoms as calculated by subtracting the rating before the CO_2_ inhalation from the post rating. In healthy volunteers, multilevel models with an unstructured variance–covariance matrix were used to account for the fact that the subjects underwent four CO_2_ concentrations. A significant overall test effect was examined in more detail by testing pairwise comparisons using Holm's method to control for the family-wise error rate.^35^ Patients and healthy volunteers were compared using univariate analysis.

#### Physiological measurements: animals and humans

In rodents, ~400 breathing-induced pressure oscillations were analyzed of the second half of each gas exposure phase. Moving artifacts, coughing, sighs and sniffing were excluded. Interbreath interval, tidal volume and ventilation were calculated for all mice. The animal temperature obtained immediately after the recording was used to calculate tidal volume and ventilation, which were corrected to animal weight in grams. With regard to electrocardiogram data, 30 s were analyzed of the second half of each gas exposure phase. Electrocardiogram data were further analyzed using the quick peaks gadget in Origin 9.0 (OriginLab, Northampton, MA, USA).

In humans, physiological data were averaged to the means per second. The 10 s before the inhalation were taken as baseline and 30 s after inhaling CO_2_ (starting after breath holding for 4 s) to assess the effects of the exposure. This period was chosen based on the observation that most individuals reach a symptomatic peak within the first 10–15 s, after which the symptoms quickly disappear. Owing to technical failure, the final sample varied per outcome and CO_2_ concentration: healthy volunteers—cardiovascular parameters *n=*117–136, respiratory outcomes *n=*47–62; PD patients—cardiovascular parameters *n=*98 (see Supplementary Table 2 for exact sample sizes).

To be able to compare the effects between mice and humans, effect sizes were calculated for physiological parameters by subtracting the mean value during air exposure from the mean value of the CO_2_ exposure, which was then divided by the s.d. of the air exposure. Effect sizes were statistically compared between conditions using *z*-tests, corrected for multiple testing.

## Results

### Behavioral response to CO_2_ exposure in mice

**Figure 1:**
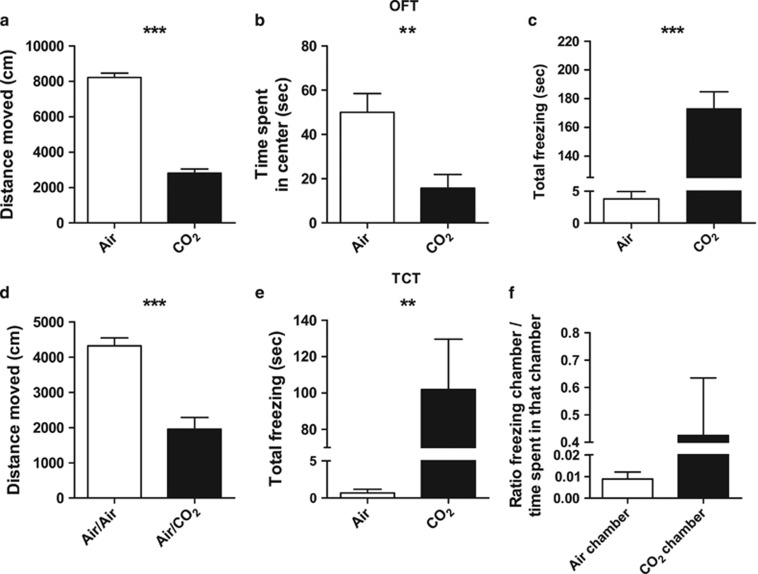
Effect of CO_2_ exposure on behavior in mice. In the open field test (OFT), CO_2_ exposure decreased the total distance moved (**a**) and the time spent in the center zone (**b**). (**c**) Under CO_2_ exposure mice froze significantly more than under air exposure. (**d**) In the two-chamber test (TCT), mice exposed to CO_2_ in one chamber covered less distance than mice exposed to air only. (**e**) CO_2_-exposed mice froze significantly more than animals exposed to air. (**f**) Correcting for the time spent in each chamber confirmed that freezing was strongest in the CO_2_ chamber, which however did not reach statistical significance. Data represent mean+s.e.m. **P<0.01, ***P<0.001.

In the OFT, mice exposed to 9% CO_2_ for 20 min covered significantly less distance (F(1, 18)=274.854, *P<*0.001, Figure 1a), spent less time in the center zone (F(1, 18)=10.610, *P=*0.004, Figure 1b) and spent more time in the corner zones in comparison with air-exposed mice (F(1, 18)=43.073, *P<*0.001, see Supplementary Table 1). Mice also showed a marked freezing response, considered to be a reflection of fear-related behavior in rodents,^36^ when exposed to CO_2_ compared with exposure to air (*t*(9.031)=−6.164, *P<*0.001, Figure 1c). Next, we used a 10 min TCT, with one chamber filled with CO_2_ and one with air, or both chambers filled with air. Overall, CO_2_ exposure decreased the distance moved when compared with air exposure only (F(1, 17)=35.826, *P<*0.001, Figure 1d). In addition, the total number of crossings between the two chambers was significantly lower in the group exposed to CO_2_ (F(1, 17)=8.080, *P=*0.011, see Supplementary Table 1). Time spent did not differ between the chamber filled with CO_2_ and the one filled with air (F(1)=0.020, *P=*0.891, see Supplementary Table 1). However, mice showed a marked freezing response when exposed to CO_2_ in one chamber compared with mice exposed to air only (*t*(8.005)=−3.656, *P=*0.006, Figure 1e). This response was particularly robust in the chamber filled with CO_2_ compared with the air chamber (F(1)=9.009, *P=*0.017, see Supplementary Table 1). As the freezing duration influences the time spent in the chambers, the ratio of freezing and time spent in a particular chamber was calculated, confirming that the freezing response was most pronounced in the CO_2_ chamber, although this did not reach statistical significance (F(1)=3.757, *P=*0.110, Figure 1f). Further, on average, the number of fecal pellets, a relative measure of anxiety/fear in rodents,^37^ significantly increased under CO_2_ exposure (F(1, 18)=12.211, *P=*0.003, see Supplementary Table 1).

### Symptom reports to CO_2_ exposure in healthy individuals and PD patients

**Figure 2:**
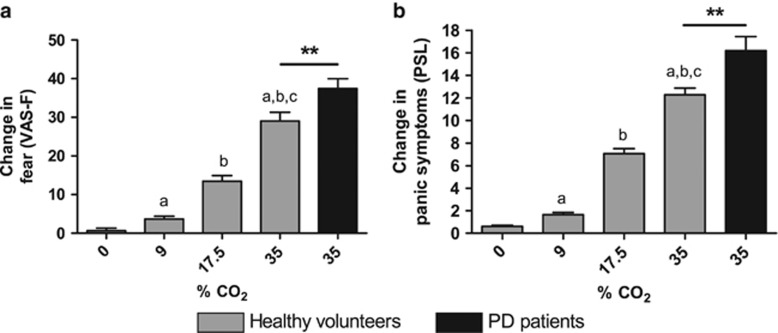
Effect of CO_2_ on self-reported fear and panic symptoms in healthy volunteers and PD patients. In healthy volunteers (gray), both fear (**a**) and panic symptoms (**b**) increased dose-dependently. Inhaling 35% CO_2_ triggered a more robust response in patients (black) when compared with healthy volunteers. Data represent mean+s.e.m. (**a**) Compared with 0% CO_2_, *P<*0.001; (**b**) compared with 9% CO_2_, *P<*0.001; (**c**) compared with 17.5% CO_2_, *P<*0.001; **P<0.01. PD, panic disorder; PSL, Panic Symptom List; VAS-F, Visual Analog Scale for fear.

In humans, we first measured the behavioral response to CO_2_ by means of a VAS-F and the PSL. In healthy individuals, taking a double vital capacity breath of four different CO_2_ concentrations up to 35% CO_2_,^6, 16^ VAS-F and PSL ratings increased dose-dependently (VAS: F(1)=227.866, *P<*0.001, PSL: F(1)=275.359). PD patients, taking a single vital capacity breath of 35% CO_2_,^8, 11^ also reported a strong fear and panic symptom response to CO_2_ (VAS: F(1)=19.078, *P<*0.001, PSL: F(1)=275.359, *P<*0.001; Figures 2a and b).

### Physiological response to CO_2_ exposure in mice, healthy volunteers and PD patients

**Figure 3:**
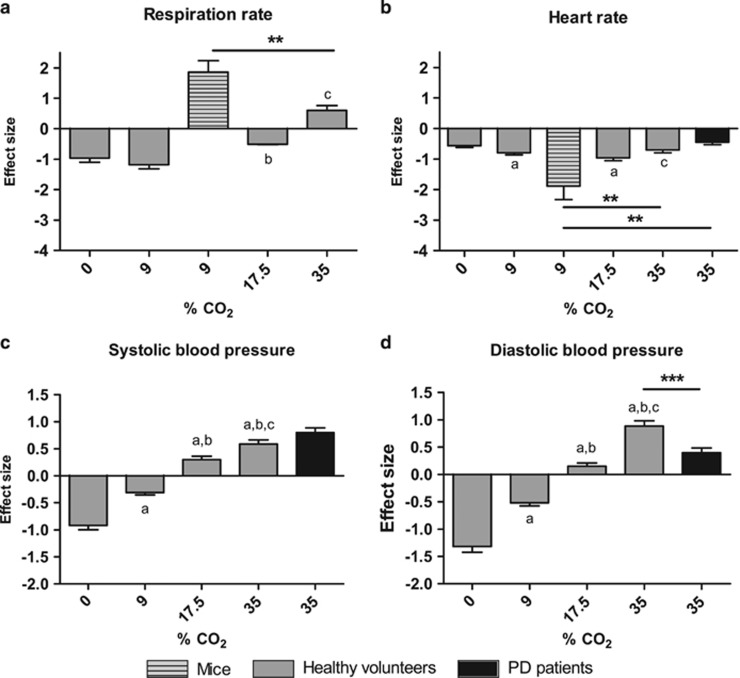
Effect of CO_2_ on respiration rate, heart rate and blood pressure in mice, healthy volunteers and panic disorder (PD) patients. (**a**) In mice (dashed), CO_2_ strongly increased the respiration rate compared to baseline (yielding a positive effect size), which was also observed in healthy volunteers (gray) after inhaling 35% CO_2_. (**b**) CO_2_ exposure decreased the heart rate in all groups, particularly in mice. (**c**, **d**) in healthy volunteers, 17.5 and 35% CO_2_ increased the blood pressure compared to baseline, which was also the case for PD patients (black) after 35% CO_2._ Data represent effect sizes+s.d. (**a**) Compared with 0% CO_2_, *P<*0.05; (**b**) compared with 9% CO_2_, *P<*0.05; (**c**) compared with 17.5% CO_2_, *P<*0.05; ***P<0.001; **P<0.01.

In a next conceptual step, toward a more objective quantitative comparison, we measured the physiological response to CO_2_ exposure (see Supplementary Table 2 for means±s.e.m.). In mice, exposure to 9% CO_2_ caused a robust increase in the respiration rate (Figure 3a, see Supplementary Table 3 for additional respiratory measurements). In healthy volunteers, no difference was found between 0 and 9% CO_2_ (*z*=1.1, *P=*0.270), and both caused a lower mean respiration rate compared with baseline (Figure 3a). At CO_2_ concentrations of 9% and higher there was a relative increase in effect size (9% compared with 17.5%: *z*=−3.61, *P<*0.001; 17.5% compared with 35%: *z*=−5.5, *P<*0.001). Only 35% CO_2_ caused an increase in the mean respiration rate compared with baseline, yielding a positive effect size. The increase in the respiration rate was more pronounced in mice after prolonged exposure to 9% than in healthy volunteers after taking a double breath of 35% CO_2_ (*z*=3.055, *P=*0.002). Regarding the heart rate, a CO_2_-induced decrease was observed across species, with a particularly strong effect in mice (compared with healthy individuals 35% CO_2_: *z*=−2.599, *P=*0.009; compared with PD patients 35% CO_2_: z=−3.160, *P=*0.002; Figure 3b). Further, in healthy volunteers, after inhaling 0 and 9% CO_2_, the mean blood pressure decreased, whereas an increase was observed after inhaling an intermediate (17.5%) and high (35%) concentration of CO_2_. Pairwise comparisons revealed significant differences between all CO_2_ concentrations regarding the systolic (0% compared with 9%: *z*=−6.78, *P*<0.001; 0% compared with 17.5%: *z*=−12.20, *P<*0.001; 0% compared with 35%: *z*=−13.84, *P<*0.001; 9% compared with 17.5%: *z*=−8.23, *P<*0.001; 9% compared with 35%: *z*=−10.46, *P<*0.001; 17.5% compared with 35%: *z*=−3.01, *P=*0.003; Figure 3c) and the diastolic blood pressure (0% compared with 9%: *z*=−6.71, *P<*0.001; 0% compared with 17.5%: *z*=−12.21, *P<*0.001; 0% compared with 35%: *z*=−15.63, *P<*0.001; 9% compared with 17.5%: *z*=−8.31, *P<*0.001; 9% compared with 35%: *z*=−12.87, *P<*0.001; 17.5% compared with 35%: *z*=−6.67, *P<*0.001; Figure 3d). On average, the effect size with regard to the systolic blood pressure tended to be higher in PD patients in comparison with healthy volunteers (*z*=1.83, *P=*0.067), whereas the effect size of the diastolic blood pressure was lower in patients than in healthy volunteers (*z*=3.89, *P<*0.001).

## Discussion

The incongruence between rodent and human experimental fear models hampers the translation of findings obtained in the two species. The present study bridges this gap by applying the same stimulus, that is, CO_2_, and obtaining a *quantitative* comparison of the same physiological outcome parameters in addition to commonly used behavioral phenotypes in three samples: in mice, healthy volunteers and PD patients. Using this approach, we showed that in both human samples CO_2_ triggers a marked fear response associated with an increase in blood pressure, an adaptive decrease in the heart rate and in healthy individuals an increase in respiration rate. A comparable behavioral and physiological response was observed in mice, demonstrating corresponding effects across species.

The use of a CO_2_ inhalation in form of a single vital capacity breath of 35% CO_2_ as human experimental model for panic was first established in PD patients.^38^ In contrast to initial assumptions,^12, 13, 14, 15^ the reactivity to CO_2_ is not limited to PD patients. Meanwhile, it has been repeatedly shown that CO_2_ induces a dose-dependent state of experiencing fear and panic symptoms in healthy individuals.^7, 39^ This observation implies that the reactivity to CO_2_ is a continuously distributed trait and suggests the existence of a sensitivity spectrum, with PD patients being at the highest end of sensitivity. This notion is supported by imaging studies.^40, 41, 42^ Here we provide, for we believe the first time, a direct quantitative comparison of the response to CO_2_ between PD patients and healthy individuals. It has been well established that inhaling CO_2_ triggers the emotional response and panic symptoms in PD patients associated with real-life PAs^5, 11^ as well as in healthy individuals complying with the formal criteria of a PA in the current DSM, when using a higher concentration.^16^ However, that comparison in healthy individuals was qualitative in nature. The quantitative comparison of the physiological response in the present study shows that several physiological outcome measurements of healthy individuals were statistically comparable to those of PD patients. This suggests that the physiological reactivity induced by a double vital capacity breath of 35% CO_2_ in healthy individuals reflects well the reactivity provoked by a single vital capacity breath of 35% CO_2_ in PD patients, thus further supporting the potential to study healthy individuals before involving patients to avoid confounding effects from comorbid psychiatric disorders or current/past treatments. However, it has to be taken into consideration that, whereas the reactivity is similar on the physiological level, PD patients have a stronger emotional response to CO_2_ than healthy individuals.

To explore the underlying basic mechanisms of panic, CO_2_ exposure has also been applied in rodents.^25, 26, 27, 28^ The assessment of the behavioral response requires a long exposure to CO_2_, which makes it unfeasible to apply the high dosage of 35% that is used in humans. The lower concentrations of CO_2_ in the healthy individuals thus serve to make the bridge to the lower CO_2_ concentrations in the animal study. Up to now it was unclear to what extent CO_2_ exposure in rodents represents a good experimental model for panic in humans. In order to determine this, the model can be judged on the criteria of face, predictive and construct validity.^3^ Increasing evidence, including the present study, supports a relatively good face validity.

CO_2_ exposure provokes a robust behavioral fear response in humans and in rodents. In humans, this response is expressed in terms of self-reported fear and panic symptoms in the present and other studies,^11, 16^ whereas in rodents it is expressed in terms of the behavioral response itself, particularly freezing is considered to reflect fear-related behavior.^36^ In the present study, we observed that, for example, the distance moved was strongly reduced in both the OFT and the TCT when animals were exposed to CO_2_ compared with air exposure. Thereby, our data confirm previous studies regarding the CO_2_-induced behavioral effects in rodents.^25, 26, 27^ However, in the TCT, in contrast to a previous report^27^ and our expectation, mice did not spend less time in the chamber filled with CO_2_ than in the chamber filled with air. When inhaling CO_2_ or during a PA, humans often attempt to avoid the aversive situation. Analogously, we expected mice to avoid the aversive effects of CO_2_. This was, however, not the case. This finding might be explained by the very strong observed freezing response that prevented the animals from leaving the chamber filled with CO_2_. In addition to the behavioral fear response, in this study, mice displayed a response that was comparable to the one in humans on the physiological level. However, the predictive validity of the rodent CO_2_ model is still to be determined. In contrast to the widespread use of CO_2_ in human panic studies, rodent studies in the framework of panic are still scarce. To evaluate the predictive validity of the rodent model, future studies could assess the effects of medication that is clinically effective in humans. For instance, selective serotonin reuptake inhibitors are often used in the treatment of PD^43^ and blunt the response to a CO_2_ inhalation in humans.^8, 44, 45, 46^ It was also shown that the decrease in the response to a CO_2_ challenge early in treatment (after 1 week) precedes and predicts the later clinical response.^45^ Selective serotonin reuptake inhibitors are generally considered as the first-line pharmacological treatment option for PD;^43^ however, it was recently reported in a systematic review that benzodiazepines appear to be superior regarding efficacy and side effects.^47^ Notably, some benzodiazepines (particularly clonazepam and alprazolam) also exert panicolytic effects on a CO_2_ inhalation.^48, 49, 50^

Thus, after pharmacological manipulation, it is expected that animals respond less to CO_2_ than without treatment. The last criterion, construct validity, is strongly supported by recent studies. On the basis of a series of experiments in mice, demonstrating an essential role of the acid-sensing ion channel (ASIC) 1a in CO_2_-induced fear behavior,^27^ genetic research in humans has made progress. Recently, an association between polymorphisms in the human homolog gene amiloride-sensitive cation channel 2, encoding the pH-sensitive ion channel, and the diagnosis of PD was reported.^51^ We investigated the effects of CO_2_ exposure as done in a seminal rodent study,^27^ showing a genetic moderation of the same polymorphisms on the emotional response in PD patients and on the physiological response in healthy individuals.^52^ Previously, we proposed that an acutely disturbed brain acid–base homeostasis represents the mechanism underlying a (CO_2_-provoked) PA. This is supported by experiments in rodents, demonstrating that CO_2_ exposure causes a drop in brain pH,^27, 53^ and evidence from intravenous bicarbonate infusions points toward the same effect in humans.^54^ A shift out of the normal physiological pH range can have life-threatening consequences. ASIC1a as a pH detector that triggers adaptive responses might therefore represent a key link between pH changes and panic behavior. In addition to ASIC1a, accumulating evidence involves orexin as another candidate involved in panic states (for review see Johnson *et al.*^55^). Orexin is produced in CO_2_/pH-sensitive hypothalamic neurons that regulate sympathetic responses and blood pressure and project to brain regions implicated in behavioral defense. Rodent research showed that disinhibiting the orexin system is associated with developing a panic-prone state, whereas administration of orexin receptor antagonists block the panic response to CO_2_ exposure and sodium lactate infusion. Furthermore, in humans, increased levels of cerebrospinal orexin were found in subjects with panic anxiety.^55^ On the basis of these studies, the etiological processes between the disorder in humans and the animal model appear to converge toward high construct validity. Taken together, judging the three validity criteria shows that the rodent CO_2_ model reflects the aspects of PD well.

A cross-species model as used in the present study can strongly facilitate the current understanding of the neural basis of a disorder. Combining various dimensions such as behavior and neurobiological measures might contribute to a more effective and etiology-based diagnosis in line with the research domain criteria framework, and eventually to new treatment options. At least in panic research, it appears that the reactivity to CO_2_ in mice can serve as a model for humans, and the reactivity in healthy individuals as a model for PD patients. Thus, new potential treatment strategies can be tested in mice and healthy individuals before eventually being offered to patients.

A few considerations and future directions should be kept in mind when interpreting the present data. First, PAs in PD occur unexpectedly, which makes it challenging to study them in real life. Therefore, we made use of a CO_2_ inhalation in the laboratory. However, this does not reflect the unexpected nature of real-life PAs. Future studies might benefit from ambulatory assessments of self-reports and physiological monitoring that have become more feasible with the development of advanced systems.^56^ Thereby, natural data and different states over the course of a day could be captured. Although newer devices represent a promising approach in this respect, the infrequency of real-life PAs might lead to long assessment periods and healthy individuals could not be studied anymore, as they do not experience naturally occurring PAs. When studying experimental PAs using CO_2_ inhalations, it might be interesting to examine potential subtypes as done in previous studies in nonclinical participants.^57, 58^ For instance, assessing whether specific symptoms are present predominantly at a particular concentration in patients might provide more in-depth insights. Second, in humans, particularly one or two vital capacity breaths of 35% CO_2_ are validated as the experimental model for PAs (PD patients and healthy individuals, respectively), which is not readily feasible to apply in mice. Apart from studying the behavioral response, we (and others^27^) make use of a prolonged exposure to a lower percentage in mice. Although this percentage is not identical to the one in humans, the robust behavioral fear response and the physiological response being quantitatively similar to the one in humans suggests that the model represents a panic model. Future studies are needed to validate this model, also pharmacologically. Third, we did not measure blood pressure in mice, as commonly used methods such as a tail cuff do not allow assessment of freely moving animals. Telemetry might be an alternative, but requires prior surgery. Further, we did not obtain respiratory parameters in patients, as fixing the mask to the head was found unacceptable in a previous pilot study in our laboratory. Smaller, less disturbing devices might be an useful approach in future studies to be able to compare the CO_2_-induced respiratory effects between samples.

To conclude, the present project uses the same experimental stimulus as well as outcome measurements and quantitatively compares the data between mice, healthy individuals and patients to study a psychopathological phenomenon, demonstrating corresponding effects across species. This model strongly increases the efficacy to translate knowledge generated in the laboratory to human research and has a large potential to drive forward, elucidating the molecular mechanisms involved in the pathophysiology of PD and to extend basic discoveries into the daily health practice. In the long run, this might be a step on the road to a novel classification of the diagnostic criteria for PD incorporating the etiological basis, and to an improved and more personalized treatment.
