# Supplementary material for: CO2 exposure as translational cross-species experimental model for panic
Source: Transl Psychiatry. 2016 Sep 6;6(9):e885–. doi: 10.1038/tp.2016.162 (PMC5048202; doi:10.1038/tp.2016.162)
Supplement: Supplementary Information [file tp2016162x1.docx]

**Supplementary information**

**
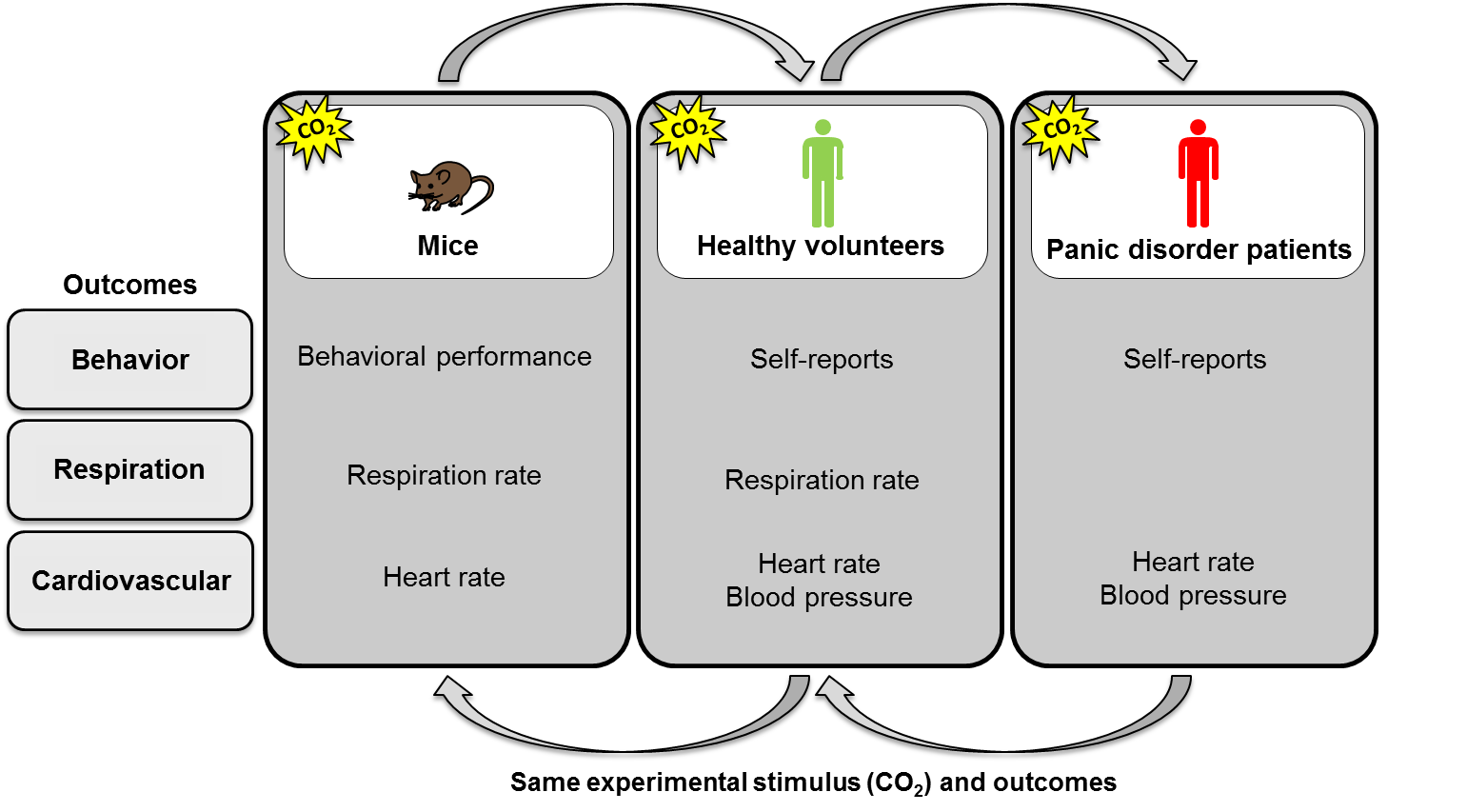
**

**Figure 1. Overview of the translational approach used in the present project.** The same experimental stimulus (carbon dioxide, CO_2_) was applied and the same cardio-respiratory outcome measurements were obtained in addition to the commonly used behavioral ones.

**Table 1. Effect of CO_2_ on various outcomes of the open field test and the two-chamber test.** Data represent mean values (± SEM).

|  | **Outcome** | **Air** | **CO_2_** | **p-value** |
| --- | --- | --- | --- | --- |
| **Open field test** | **Time spent in corners (sec)** | 682.63 ± 14.85  (n=10) | 913.24 ± 31.84  (n=10) | 0.001 |
|  | **Fecal pellets** | 5.60 ± 0.98  (n=10) | 6.2 ± 0.59  (n=10) | 0.607 |
|  | | | |  |
| **Two-chamber test** | **Distance moved per chamber**  (cm; mice exposed to CO_2_/air; n=9) | 1317.89 ± 318.33 | 635.78 ± 105.04 | 0.078 |
|  | **Number of crossings** (n=9) | 7.3 ± 1.37 | 2.56 ± 0.88 | 0.011 |
|  | **Time spent in each chamber**  (sec; mice exposed to CO_2_/air; n=9) | 304.02 ± 72.49 | 283.13 ± 74.94 | 0.891 |
|  | **Freezing per chamber**  (sec; mice exposed to CO_2_/air; n=9) | 96.89 ± 29.08 | 5.00 ± 2.42 | 0.017 |
|  | **Fecal pellets** (n=9) | 1.8 ± 0.76 | 5.20 ± 0.61 | 0.003 |
|  | **Fecal pellets per chamber**  (mice exposed to CO_2_/air; n=9) | 2.7 ± 0.78 | 2.5 ± 0.77 | 0.891 |
|  |  | | |  |
|  |  | **Left air chamber** | **Right air chamber** | **p-value** |
|  | **Distance moved per chamber**  (cm; mice exposed to air only; n=10) | 2402.92 ± 211.77 | 1912.58 ± 345.21 | 0.376 |
|  | **Time spent in each chamber**  (sec; mice exposed to air only; n=10) | 326.80 ± 41.57 | 249.39 ± 43.17 | 0.385 |
|  | **Freezing**  (sec; mice exposed to air only; n=10) | 0.50 ± 0.40 | 0.20 ± 0.13 | 0.394 |
|  | **Fecal pellets per chamber**  (mice exposed to air only; n=10) | 1.10 ± 0.53 | 0.70 ± 0.34 | 0.399 |

**Table 2. Effect of CO_2_ on respiration rate, heart rate, and blood pressure.** Data represent mean values (± SEM).

|  |  | **Mice** | | **Healthy volunteers** | | **Panic patients** | |
| --- | --- | --- | --- | --- | --- | --- | --- |
| **Outcome** | **% CO_2_** | **Baseline** | **CO_2_** | **Baseline** | **CO_2_** | **Baseline** | **CO_2_** |
| **Respiration rate** | 0 |  |  | 16 ± 0.62  (n=59) | 11 ± 1.34  (n=59) |  |  |
|  | 9 | 263 ± 14.64  (n=19) | 382 ± 13.68  (n=19) | 16 ± 0.56  (n=61) | 11 ± 1.47  (n=61) |  |  |
|  | 17.5 |  |  | 16 ± 0.60  (n=47) | 14 ± 1.80  (n=47) |  |  |
|  | 35 |  |  | 16 ± 0.50  (n=53) | 18 ± 2.38  (n=53) |  |  |
| **Heart rate** | 0 |  |  | 91 ± 1.43  (n=127) | 82 ± 3.37  (n=127) |  |  |
|  | 9 | 661 ± 13.47  (n=18) | 554 ± 20.16  (n=18) | 91 ± 1.37  (n=135) | 78 ± 3.25  (n=135) |  |  |
|  | 17.5 |  |  | 91 ± 1.43  (n=118) | 76 ± 2.72  (n=118) |  |  |
|  | 35 |  |  | 90 ± 1.27  (n=119) | 80 ± 3.30  (n=119) | 95 ± 1.97  (n=98) | 86 ± 1.66  (n=98) |
| **Systolic blood pressure** | 0 |  |  | 129 ± 1.43  (n=126) | 115 ± 4.17  (n=126) |  |  |
|  | 9 |  |  | 134 ± 1.58  (n=132) | 128 ± 4.70  (n=132) |  |  |
|  | 17.5 |  |  | 131 ± 1.61  (n=117) | 136 ± 5.15  (n=117) |  |  |
|  | 35 |  |  | 125 ± 1.76  (n=117) | 137 ± 5.52  (n=117) | 137 ± 1.97  (n=98) | 153 ± 2.50  (n=98) |
| **Diastolic blood pressure** | 0 |  |  | 78 ± 0.77  (n=126) | 67 ± 3.03  (n=126) |  |  |
|  | 9 |  |  | 80 ± 0.92  (n=132) | 75 ± 3.30  (n=132) |  |  |
|  | 17.5 |  |  | 79 ± 0.94  (n=117) | 81 ± 3.48  (n=117) |  |  |
|  | 35 |  |  | 76 ± 0.78  (n=117) | 84 ± 3.70  (n=117) | 83 ± 0.99  (n=98) | 87 ± 1.12  (n=98) |

**Table 3. Effect of CO_2_ on inter-breath interval, tidal volume, and ventilation in mice.** Data represent mean values (± SEM; tidal volume and ventilation corrected for body weight; n=19 for all outcome measurements).

| **Outcome** | **Baseline** | **CO_2_** | **p-value** |
| --- | --- | --- | --- |
| **Inter-breath interval (s)** | 0.26 ± 0.01 | 0.16 ± 0.01 | <0.001 |
| **Tidal volume (ml/g)** | 0.06 ± 0.00 | 0.10 ± 0.00 | <0.001 |
| **Ventilation (ml/g/min)** | 13.37 ± 1.29 | 37.97 ± 2.63 | <0.001 |
